# P04.50. Acupuncture for lumbar spinal stenosis: a systematic review

**DOI:** 10.1186/1472-6882-12-S1-P320

**Published:** 2012-06-12

**Authors:** K Kim, S Noh, B Lee, J Kim, G Yang

**Affiliations:** 1 School of Korean Medicine, Pusan National University, Yangsan, Republic of Korea

## Purpose

This study aims to evaluate evidence indicating the effectiveness and safety of acupuncture for lumbar spinal stenosis (LSS).

## Methods

We researched five English databases (EMBASE, MEDLINE, CENTRAL, CINAHL and AMED) and one Chinese database (CAJ) in November 2011, without language restriction. Randomized controlled trials (RCTs) of needle acupuncture for LSS were eligible.

## Results

Of 237 initially located articles, three RCTs conducted in China on 336 patients in total were included. Because of high or uncertain risk of bias (particularly in domains including allocation concealment, patient or assessor blinding and selective outcome reporting) data could not be combined in a meta-analysis. All three RCTs measured how many patients had improved. One compared deep needling at Jiaji (Ex-B2) to conventional acupuncture, showing greater benefit from deep needling after treatment (RR 1.09; 95% CI 1.01 to 1.17; p=0.023) and at three-month follow-up (RR 1.14; 95% CI 1.02 to 1.27; p=0.016), respectively. The second, comparing electro-acupuncture in combination with bloodletting therapy to electro-acupuncture alone, presented more benefit from combined therapy at post-treatment (RR 1.27; 95% CI 1.01 to 1.61; p=0.045). The third compared manual acupuncture plus Chinese herbal medicine to acupuncture alone, demonstrating more favorable results for manual acupuncture plus herbal medicine (RR 1.19; 95% CI 1.02 to 1.39; p=0.025), along with better scores on overall clinical improvements (WMD 3.20; 95% CI 1.32 to 5.08, p=0.001) after treatment. None of these studies measured the patient’s quality of life or reported on adverse events.

## Conclusion

There is insufficient evidence for, or against, using acupuncture for LSS. The benefits reported here should be interpreted with caution because of high or uncertain risk of bias in each study. The safety of acupuncture for LSS remains unclear because of the lack of reporting on adverse events. Future trials should be conducted, using rigorous methodology, appropriate comparison and clinically relevant outcomes.

